# Pathogenicity and Genomic Characterization of *Pasteurella multocida* Serogroup F Isolate AH01 From Porcine Pneumonia in China

**DOI:** 10.1155/tbed/9979547

**Published:** 2025-11-11

**Authors:** Yang-Yang Li, Hai-Xia Li, Chuan-Hao Fan, Hui-Qiang Zhen, Ye-Qing Zhu, Shouyu Wang, Guang Zhang, Gairu Li

**Affiliations:** ^1^College of Animal Science, Anhui Science and Technology University, Chuzhou, China; ^2^Anhui Engineering Technology Research Center of Pork Quality Control and Enhance, Anhui Science and Technology University, Chuzhou, China; ^3^OptiX+ Laboratory, Wuxi University, Wuxi, China; ^4^Vaccine R&D Center, Qilu Animal Health Products Co., Ltd., Jinan, China; ^5^State Key Laboratory for Animal Disease Control and Prevention, South China Agricultural University, Guangzhou, China

**Keywords:** genetic characteristics, genome annotation, *Pasteurella multocida* serogroup F, pathogenicity, whole-genome sequencing

## Abstract

*Pasteurella multocida* (*P. multocida*), a significant animal pathogen, causes swine pneumonia and atrophic rhinitis, primarily associated with serogroups A, D, and F. Although serogroups A and D are prevalent in pigs and well-established causes of these diseases, the pathogenicity and genomic characteristics of porcine serogroup F remain poorly characterized. Here, we isolated a virulent *P. multocida* strain—AH01, from pigs with fatal acute respiratory disease in Anhui, China. It was characterized as a capsular Type F, lipopolysaccharide (LPS) antigen Type L3 isolate of sequence type (ST) 9. To evaluate the pathogenicity of this strain, pigs were challenged intratracheally with AH01 (6 × 10^9^ CFU), inducing acute pyrexia, dyspnea, anorexia, and rapid mortality (≤12 h postinfection, hpi). PacBio SMRT (Single-Molecule Real Time) sequencing generated a complete 2.27-Mbp chromosome (40.3% GC content; 2058 CDSs). Annotation identified 254 potential virulence-associated genes, 47 different drug resistance phenotypes, and three genomic islands (GIs). Comparative genomics revealed a novel 16.7-kb specific region insertion encoding zonula occludens toxin (Zot) and general secretion pathway protein D (GspD), potentially facilitating epithelial barrier disruption. Furthermore, polymorphisms in LPS outer core biosynthesis genes *natC* and *gatF* were characterized across strains avian Pm70, porcine AH01, and HN07. Strain AH01 harbors a single-nucleotide deletion (*natC* position 760), causing a frameshift and premature stop. Both porcine strains AH01 and HN07 exhibited a 216-bp N-terminal extension in *gatF* compared to avian Pm70 strain, indicating host-specific or strain-dependent LPS biosynthetic divergence. Collectively, these findings provide critical insights into the pathogenicity and genomic basis of porcine-derived *P. multocida* serogroup F.

## 1. Introduction

*Pasteurella multocida* (*P. multocida*), a gram-negative facultative anaerobe, imposes substantial economic burdens on global livestock industries through syndromes including fowl cholera in poultry, hemorrhagic septicemia in cattle, and respiratory infections (rhinitis/pneumonia) in rabbits, as well as progressive atrophic rhinitis and the respiratory disease complex in swine [1–4]. This pathogen exhibits significant antigenic diversity, classified into five capsular serogroups (A, B, D, E, and F) and 16 lipopolysaccharide (LPS) antigen serotypes, which dictate host specificity and disease manifestations [5, 6].

Serogroup F of *P. multocida* was initially reported in turkeys in the United States in 1987 and has since been predominantly associated with avian hosts, serving as a primary etiological agent in fowl cholera outbreaks [7, 8]. Beyond avian species, this serogroup demonstrates significant cross-species adaptability, with documented infections in swine, sheep, cattle, and rabbits [9–13]. In swine, serogroup F was first identified in 2003 [14]. Subsequently, it has been consistently isolated from pigs with pneumonia in China since 2015, and this clinical association is further supported by experimental evidence from Peng et al. [15], who established a causal relationship between serogroup F and porcine pneumonia [16]. Notably, from 2021–2023, serogroup F was isolated from the lung tissues of fattening pigs that had succumbed to respiratory diseases on large-scale swine farms of central and eastern China, with an isolation rate of 3% [17]. Although this prevalence appears relatively low, the persistent detection and confirmed pathogenicity of serogroup F suggested that serogroup F is an emerging respiratory pathogen of clinical relevance in Chinese swine populations. However, studies on the pathogenicity and genomic characteristics of porcine-derived serogroup F isolates remain scarce, hindering a comprehensive understanding of its role in swine respiratory disease.

Whole-genome sequencing (WGS) has revolutionized bacterial pathogenesis research, offering unprecedented resolution for analyzing phylogenetic relationships, evolutionary trajectories, virulence determinants, antimicrobial resistance (AMR) profiles, and host-specific adaptations [18, 19]. The high-fidelity assemblies achievable with third-generation sequencing technologies, such as PacBio SMRT (Single-Molecule Real Time) sequencing, are particularly valuable for resolving complex genomic regions, including repetitive elements, mobile genetic elements (MGEs) like genomic islands (GIs) and prophages, and intricate gene clusters involved in capsule and LPS biosynthesis. While WGS data for *P. multocida* isolates from various hosts (avian, bovine, ovine, and lagomorph) and serogroups are increasingly available in public repositories like the NCBI Genome database [1, 20], a striking disparity exists for porcine serogroup F strains. Until now, only three complete genome sequences for *P. multocida* serogroup F isolates from swine were publicly accessible. This severe limitation constrains comparative genomic analyses, functional annotation efforts, and ultimately, our capacity to elucidate the genetic basis underlying the pathogenicity and host tropism of these emerging porcine pathogens.

In December 2022, an acute respiratory disease outbreak affected a large commercial swine farm in Anhui Province, China. Bacteriological analysis of lung samples consistently identified *P. multocida* as the causative agent. Capsular serogroup typing of a representative lung isolate (AH01) confirmed its classification as serogroup F. The persistent detection of serogroup F strains in Chinese swine herds, including this outbreak and previous reports [3, 21], suggested that this serogroup is not merely an incidental spillover event, but represents an established and potentially evolving pathogen within pig populations. However, comprehensive genomic and pathogenic data for porcine serogroup F isolates remain scarce. To address this knowledge gap, we aimed to: (1) determine its capsular serogroup, LPS antigen type, and multilocus sequence typing (MLST); (2) characterize the pathogenicity of AH01 strain using an intratracheal porcine challenge model; (3) generate a complete and high-fidelity genome assembly using PacBio SMRT sequencing to resolve repetitive regions and MGEs; (4) identify virulence factors and AMR genes; (5) perform comparative genomics to elucidate unique genetic features, particularly novel insertions potentially associated with pathogenicity. This study characterizes the pathogenicity and genomic features of the porcine-derived *P. multocida* serogroup F strain AH01, thereby elucidating the molecular basis of its virulence and expanding knowledge on the genetic diversity within understudied serogroups.

## 2. Materials and Methods

### 2.1. Ethical Declarations

All procedures complied with institutional requirements and local legislation and were approved by the Animal Experiment Ethics Committee of Anhui Science and Technology University (Approval Number 2025082). All animal procedures adhered to ethical guidelines and prioritized the welfare of the animals used in this study.

### 2.2. Isolation and Identification of *Pasteurella multocida*

*Pasteurella multocida* AH01 strain was isolated from porcine lung tissue collected aseptically during necropsy from a fatal case of confirmed pneumonia. Primary isolation was performed on tryptic soy agar (TSA) supplemented with 5% (*v*/*v*) newborn calf serum (NBCS; Zhejiang Sijiqing Biological Engineering Materials Co., Ltd., Hangzhou, China) and incubated at 37°C for 16–18 h. Three morphologically distinct colonies were selected for purification and subjected to gram staining according to standard procedures. Microscopic examination revealed gram-negative and coccobacillary cells (0.5–1.2 μm in diameter) exhibiting typical pink staining. Selected colonies were cultured in tryptic soy broth (TSB) containing 5% NBCS and incubated aerobically with agitation (180 rpm) at 37°C for 12–16 h to achieve log-phase growth [22].

Genomic DNA was extracted using the EasyPure Bacteria Genomic DNA Kit (TransGen Biotech, Beijing, China) according to the manufacturer's instructions. Species identification was confirmed by amplification and sequencing of the *P. multocida*-specific *kmt1* gene following established methods [5]. Capsular serotype and LPS antigen type were characterized by PCR using previously described protocols [5, 6], with detailed primer information provided in Supporting Information 1: Table S1.

### 2.3. MLST

*Pasteurella multocida* isolate AH01 was characterized by MLST following the RIRDC MLST scheme [23]. PCR amplifications of the seven housekeeping genes (*adk*, *est*, *pmi*, *zwf*, *mdh*, *gdh*, and *pgi*) were performed using primers and protocols available in the RIRDC MLST scheme [23–25]. PCR products were commercially sequenced (General Biotechnology Co., Ltd., AnHui, China). Sequences of the seven loci were aligned against the corresponding reference sequences in the PubMLST database (https://pubmlst.org/) to determine allelic profiles. Sequence types (STs) were assigned by submitting allelic profiles to the PubMLST database. A neighbor-joining phylogenetic tree based on concatenated sequences was constructed using MEGA software (v11.0.13) with 1000 bootstrap replicates [26]. Details of reference *P. multocida* strains are provided in Supporting Information 1: Table S2.

### 2.4. Antibiotic Sensitivity Testing

Antimicrobial susceptibility profiles of the strains against 23 antimicrobial agents were determined using the disk diffusion method on Mueller–Hinton (MH) agar supplemented with 5% NBCS [27]. Bacterial suspensions were adjusted to a 0.5 McFarland turbidity standard (Shanghai Xinrui Instrumentation Co., Ltd.). Sterile cotton swabs were immersed in the standardized suspensions and used to inoculate agar plates uniformly to form a bacterial lawn. The plates were incubated at 37 °C for 24 h. Inhibition zone diameters were measured using digital calipers (DEGUOMNT) and interpreted according to the guidelines provided by the manufacturer (Hangzhou Biotest Biotech), CLSI M100-S33 [28], and EUCAST (v13.0) (https://www.eucast.org/clinical_breakpoints). Based on consensus criteria, the strains were categorized as susceptible (S), intermediate (I), or resistant (R).

### 2.5. Experimental Animal Infection

Nine healthy 30-day-old pigs from a commercial farm in Anhui Province were randomly allocated into two groups: challenge group (*n* = 6) and negative control group (*n* = 3). All animals were housed in separate isolation units under the same environmental conditions (22 ± 2°C, 50%–60% humidity) with ad libitum access to feed and water. Prior to challenge, nasal swabs and serum samples from all pigs tested negative for *P. multocida* by species-specific PCR (targeting the *kmt1* gene) and ELISA [29], respectively. Challenge group pigs were inoculated intratracheally with 3 mL of *P. multocida* strain AH01 suspension (2 × 10^9^ CFU/mL in sterile PBS), while negative control pigs received an equal volume of sterile PBS via the same route [30]. Clinical signs (including body temperature, respiratory rate, mental status, and feeding behavior) were recorded twice daily. Upon mortality of the last challenge group pig, surviving negative control group pigs were humanely euthanized following American Veterinary Medical Association (AVMA) guidelines. Euthanasia was performed using a two-step protocol: (1) sedation with telazol (4 mg/kg, intramuscular) to minimize distress, followed by (2) intravenous administration of sodium pentobarbital (150 mg/kg). Death was confirmed by absence of corneal reflex and palpable heartbeat. Necropsy was performed within 15 min to collect cardiac, hepatic, splenic, and pulmonary tissues for analysis. For bacterial detection, tissue homogenates were subjected to *P. multocida*-specific PCR amplification (*kmt1* gene). For histopathology, tissues were fixed in 4% neutral buffered formalin, processed through graded alcohols, embedded in paraffin, sectioned at 4–5 μm thickness, and stained with hematoxylin and eosin (H&E) for microscopic evaluation [31].

### 2.6. Genome Sequencing and Analysis

The complete genome of *P. multocida* strain AH01 was sequenced using a hybrid approach combining Illumina NovaSeq PE150 (2 × 150 bp; General Biotechnology Co., Ltd., AnHui, China) and PacBio Sequel II platforms, with achieved sequencing depths of approximately 936x and 165x, respectively. Raw Illumina reads were quality-filtered using Fastp (v0.23.2) with the following parameters: adapter trimming, minimum *Q*-score of 20, and minimum read length of 50 bp [32]. PacBio HiFi circular consensus sequencing (CCS) reads were processed with SMRT Link toolkit to retain high-fidelity reads (accuracy ≥99%, minimum pass count = 3). A hybrid de novo assembly strategy was implemented, combining third-generation sequencing data assembled with Hifiasm (v0.13-r308) and Canu (v1.7), followed by four rounds of error correction using Pilon (v1.24) with high-quality Illumina short reads (2 × 150 bp) [33]. Remaining gaps (>100 bp) were closed using GapFiller (v2.1.1) [34], followed by 10 iterations of polishing with Pilon (v1.24) to correct single nucleotide polymorphisms (SNPs) and indels (<50 bp). Assembly quality was assessed using QUAST (v5.2.0).

Functional annotation of protein-coding genes were performed via BLASTp (BLAST + v2.13.0) against the Conserved Domain Database (CDD) (https://www.ncbi.nlm.nih.gov/Structure/cdd/wrpsb.cgi), Clusters of Orthologous Groups (COG) (https://www.ncbi.nlm.nih.gov/research/cog-project/), Nonredundant (NR) (https://pubmlst.org/static/analysis/nrdb.shtml), and CluSTr (Clusters of SWISS-PROT and TrEMBL proteins) database (http://www.ebi.ac.uk/clustr/). Carbohydrate-active enzymes (CAZys) annotations were generated using HMMER3 alignments against the CAZy database (https://www.cazy.org/). Gene Ontology (GO) terms were assigned based on CluSTr best hits, while KEGG pathway mapping was performed via the KAAS server (https://www.genome.jp/kegg/kaas/) using bidirectional best-hit methods [35]. Virulence genes and antibiotic resistance genes were identified through homology searches against the Pathogen–Host Interaction Database (PHI-base; http://www.phi-base.org/) [36], the Virulence Factor Database (VFDB; https://www.mgc.ac.cn/VFs/) [37], and the Comprehensive Antibiotic Resistance Database (CARD) (https://card.mcmaster.ca/) [38], respectively. Lipoprotein prediction was performed using SignalP (v5.0) (http://www.cbs.dtu.dk/services/SignalP/) and phage/prophage regions were predicted with PHAST (http://phast.wishartlab.com/) [39]. The GIs in the genome were determined using the IslandViewer 4 (https://www.pathogenomics.sfu.ca/islandviewer) [40].

Comparative genomic analysis was conducted using two complementary approaches: (1) whole-genome alignment using progressiveMauve (v2.4.0) with default parameters [41, 42]; (2) comparative analysis of the capsule locus and the LPS outer core biosynthesis gene cluster, were visualized using Easyfig (v2.2.5) [43, 44].

## 3. Results

### 3.1. Isolation, Identification, and Typing of *Pasteurella multocida* AH01

**Figure 1:**
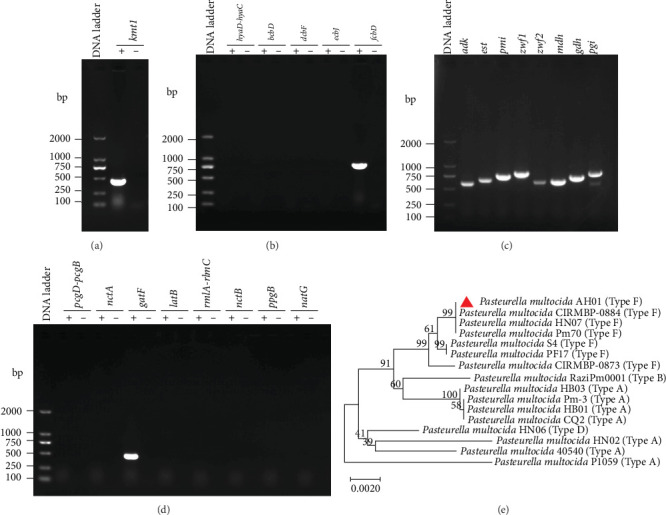
Molecular characterization of *Pasteurella multocida* isolate. (A) PCR amplification of the species-specific *kmt1* gene (460 bp) (B) Capsular serotyping PCR. Target genes: *hyaD-hyaC*, *bcbD*, *dcbF*, *ecbJ*, and *fcbD* (Type F, 851bp). (C) Multilocus sequence typing (MLST) PCR amplification. Lanes: 1, *adk*; 2, *est*; 3, *pmi*; 4, *zwf1*; 5, *zwf2*; 6, *mdh*; 7, *gdh*; 8, *pgi*. *M* = 2000 bp DNA ladder. (D) Lipopolysaccharide (LPS) antigen typing PCR. Genes as following: *pcgD-pcgB*, *nctA*, *gatF* (Type L3, 474 bp), *latB*, *rmlA-rlmc*, *nctB*, *ppgB*, and *natG*. *M* = 2000 bp DNA ladder. The symbol “+” represent positive for different genes, and “–” represent negative control. (E) Neighbor-joining phylogenetic tree of isolate AH01 based on concatenated sequences of seven MLST loci (*adk*, *aroA*, *deoD*, *gdhA*, *g6pd*, *mdh*, and *pgi*). Red triangles mark the strains isolated in this study. The phylogenetic tree was constructed using MEGA (v11.0.13) with 1000 bootstrap replicates.

Following 16–18 h incubation at 37°C, the TSA agar plate exhibited regular, circular, smooth, translucent, and grayish-white colonies, while the blood agar plate displayed small, gray, nonhemolytic colonies (Supporting Information 2: Figure S1A, B). Microscopic examination of the isolated colonies revealed gram-negative coccobacilli with rounded ends (Supporting Information 2: Figure S1C). Species-specific identification via amplification of the *kmt1* gene fragment confirmed the isolate as *P. multocida* (Figure 1A), and was named as AH01 (GenBank accession number: CP178495). PCR–based capsular and LPS serotyping of *P. multocida* AH01 identified characteristic fragments of 851 bp (capsular serotype F) and 474 bp (LPS antigen type L3), as shown in Figure 1B,D, respectively. MLST targeting seven housekeeping genes (*adk*, *est*, *pmi*, *zwf*, *mdh*, *gdh*, and *pgi*) determined the allelic profile of the strain as 2–7–5–6–4–5–7, corresponding to ST9 (Figure 1C).

Phylogenetic analysis based on seven core housekeeping genes (*adk*, *est*, *pmi*, *zwf*, *mdh*, *gdh*, and *pgi*) showed that *P. multocida* AH01 is closest affinity to Pm70 and HN07, and the serogroup F strains form a monophyletic clade (Figure 1E). Collectively, our serological and genetic analyses determined that *P. multocida* strain AH01 belongs to capsular serogroup F, possesses LPS antigen type L3, and is classified as ST9 by MLST.

### 3.2. Antimicrobial Susceptibility Test

Antimicrobial susceptibility profiling revealed that *P. multocida* AH01 exhibited sensitivity to 10 antimicrobial agents, including florfenicol, spectinomycin, and enrofloxacin; demonstrated resistance to erythromycin, lincomycin, trimethoprim, and co-trimoxazole; showed intermediate susceptibility to streptomycin, ceftriaxone, and ciprofloxacin, among others, as detailed in Supporting Information 1: Table S3.

### 3.3. Pathogenicity of *Pasteurella multocida* AH01 in Pigs

**Figure 2:**
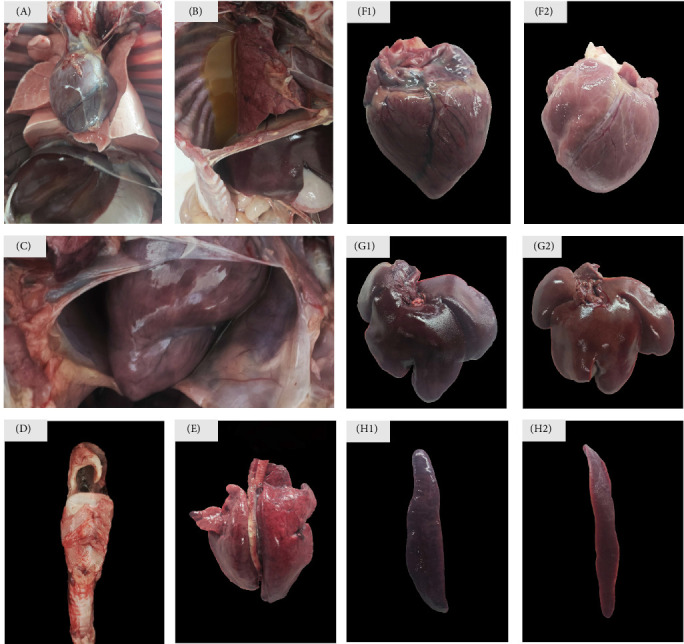
Pathological lesions in pigs infected with *Pasteurella multocida* isolate AH01. (A) Thoracic cavity, healthy control. (B) Thoracic cavity, infected pig. (C) Pericardium, infected pig. (D) Trachea, infected pig. (E) Lung (infected). (F) Heart: (F1) infected and (F2) healthy control. (G) Liver: (G1) infected and (G2) healthy control. (H) Spleen: (H1) infected and (H2) healthy control.

To assess the pathogenicity of *P. multocida* AH01, pigs were intratracheally challenged with 6.0 × 10^9^ CFU of the AH01 isolate. All negative control group pigs survived the experimental period without exhibiting clinical signs of disease, including pyrexia (body temperature <39.5°C), respiratory distress, or behavioral abnormalities. In contrast, challenge group pigs developed pronounced clinical manifestations starting at 6 h postinoculation (hpi), characterized by pyrexia (40.3–41.5°C), lethargy, tachypnea, dyspnea, and anorexia. Initial deaths in challenged pigs occurred at 12 hpi, presenting with severe mucosal cyanosis and diffuse petechial hemorrhages in the cervical/abdominal regions. The remaining fatalities among the pigs, succumbed to acute respiratory failure, were observed between 86 and 98 hpi. Gross pathological examination revealed systemic lesions consistent with acute pasteurellosis, including serofibrinous pleural effusion (Figure 2B), pericardial effusion (Figure 2C), copious frothy exudates within the tracheal lumen (Figure 2D), pulmonary consolidation characterized by severe congestion, edema, and multifocal hemorrhagic foci (Figure 2E), cardiac congestion with pronounced epicardial hyperemia and interstitial hemorrhage (Figure 2F1), hepatic congestion accompanied by centrilobular necrosis (Figure 2G1), and splenic congestion with multifocal hemorrhagic infarcts (Figure 2H1). In contrast, negative control group organs exhibited normal gross and histological morphology (Figure 2A, F2, G2, H2). These findings conclusively demonstrated the pathogenicity of *P. multocida* AH01 in porcine.

**Figure 3:**
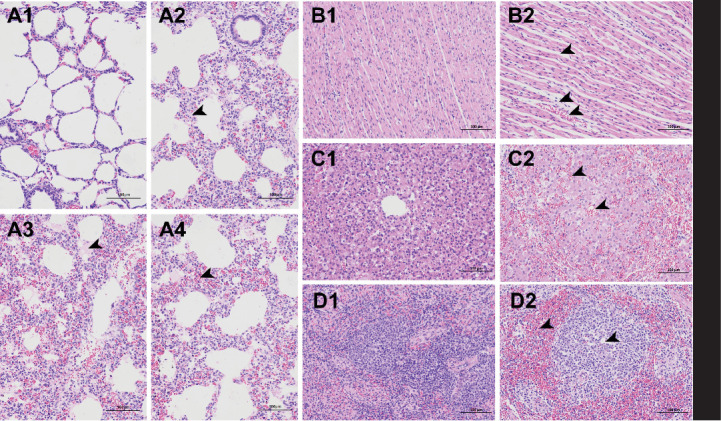
Histopathological lesions in pig challenged with *Pasteurella multocida* isolate AH01. (A) Lung: (A1) Healthy control and (A2) infected. The arrow indicates interlobular septal thickening and prominent inflammatory cell infiltration in the lung tissue of infected pigs. (A3) Infected. The arrow indicates the deposition of fibrinous exudate in the lungs of infected pigs. (A4) Infected. The arrow indicates hemorrhagic foci in the lungs of infected pigs. (B) Heart: (B1) Healthy control and (B2) infected. Arrows indicate rupture of cardiac muscle fibers, widening of the interstitium, and infiltration of numerous inflammatory cells and red blood cells in the heart tissue. (C) Liver: (C1) Healthy control and (C2) infected. Arrows indicate congestion of the central veins and hepatic sinusoids in the liver lobules, significant accumulation of exudate around the sinusoids, and marked widening of the interstitial spaces between the hepatic cords. (D) Spleen: (D1) Healthy control and (D2) infected. Arrows indicate aggregation of red blood cells, infiltration of inflammatory cells, atrophy of the white pulp, and partial regression of necrosis in the spleen. Tissues stained with hematoxylin and eosin. Scale bars: 100 μm.

Histopathological analysis further confirmed that *P. multocida* AH01 induced lesions. Lung tissues exhibited pulmonary lesions characterized by fibrinous exudate deposition, hemorrhagic foci, interlobular septal thickening, and prominent inflammatory cell infiltration (Figure 3A2,A3,A4). Cardiac tissues showed myocardial fiber fragmentation, interstitial widening, and substantial infiltration of both inflammatory cells and erythrocytes (Figure 3B2). Hepatic architecture demonstrated centrilobular venous and sinusoidal congestion, extensive perisinusoidal exudate accumulation, and marked widening of hepatic cord interstitial spaces (Figure 3C2). Splenic pathology featured erythrocyte pooling, inflammatory cell infiltration, white pulp atrophy, and partial necrotic resolution (Figure 3D2). No pathological alteration was detected in the negative control group (Figure 3A1, B1, C1, D1). Bacteriological, pure *P. multocida* AH01 colonies were successfully reisolated from challenge group pigs across multiple tissues, including tracheal mucus, cardiac tissue, pulmonary parenchyma, liver, pericardial effusion, and blood (*n* = 3 per site), data not shown. Negative control group pigs exhibited no *P. multocida* colonization, clinical signs, or gross lesions throughout the study.

### 3.4. Genomic Characterization of *Pasteurella multocida* AH01

**Figure 4:**
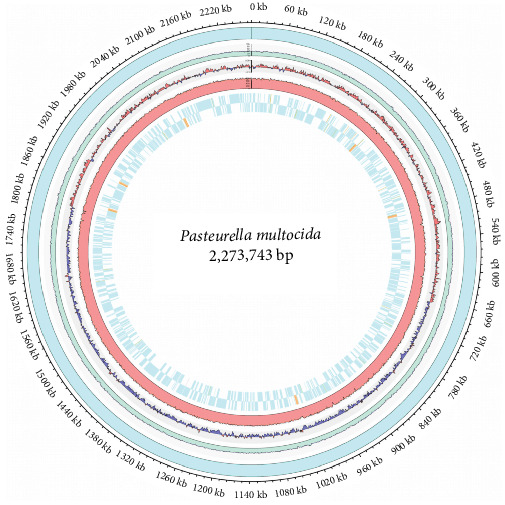
Circular genome map of *Pasteurella multocida* isolate AH01. The circles represent the following (from outside to inside: circle 1–5): circle 1 is the entire circular genome contig (cyan), circle 2 is the GC content (green), circle 3 is the GC skew: AT-rich (light blue); GC-rich (light pink), circle 4 represents the read depth (pink), and circle 5 represents the gene category: CDS (cyan), pseudo (purple), tRNA (orange), and rRNA (brown).

The *P. multocida* AH01 genome generated 38,764 raw reads (total: 377,118,100 bp; average read length: 9728.57 bp; Supporting Information 1: Table S4). The final polished assembly resolved a single circular chromosome (2,273,743 bp; GC content: 40.34%; GenBank accession number: CP178495; Figure 4) and one plasmid (4789 bp; GenBank accession number: CP178496). The raw Illumina and PacBio sequencing data are available in the NCBI SRA database under accession number SRR35394647. Annotation of the chromosome identified 2058 protein-coding sequences (CDSs), 57 tRNA genes, and 19 rRNA operons comprising six 16S rRNA, six 23S rRNA, and seven 5S rRNA genes (Supporting Information 1: Table S4).

**Figure 5:**
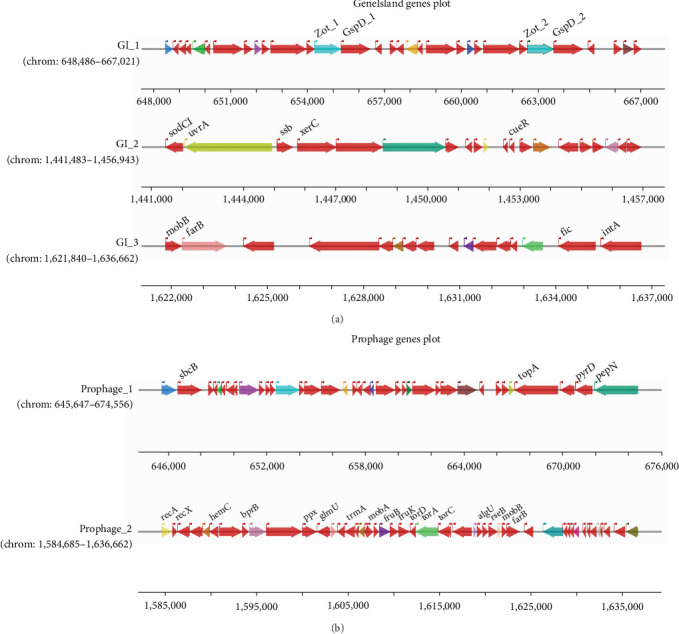
Genomic islands (GIs) and prophage regions in *Pasteurella multocida* isolate AH01. (A) Location and annotation of three GIs: GI_1 (648,486–667,021 bp), GI_2 (1,441,483–1,456,943 bp), and GI_3 (1,621,840–1,636,662 bp). (B) Identification of two intact prophages: prophage_1 (645,647–674,556 bp) and prophage_2 (1,584,685–1,636,662 bp). Genes are color-coded by functional category. Phage/prophage regions were predicted with PHAST (http://phast.wishartlab.com/). The GIs in the genome were determined using the webservers of IslandViewer 4 (https://www.pathogenomics.sfu.ca/islandviewer/).

Three GIs were identified: GI-1 (18,536 bp; 31 genes), GI-2 (15,461 bp; 20 genes), and GI-3 (14,823 bp; 16 genes; Figure 5A and Supporting Information 1: Table S5). GI-1 encodes zonula occludens toxin (Zot) and general secretion pathway protein D (GspD), GI-2 harbored the virulence gene Gifsy-2 prophage-associated Cu–Zn superoxide dismutase precursor (*sodCI*), while GI-3 contained a two-component response regulator (*farB*). Specifically, Zot can lead to the disassembly of intercellular tight junctions, while GspD, as the outer membrane channel of the bacterial type II secretion system (T2SS), secretes various toxins that induce severe diseases. Prophage prediction identified two intact regions: prophage-1 (51,978 bp; 49 genes) and prophage-2 (28,910 bp; 37 genes). Prophage-2 carried three virulence-associated genes: two-component response regulator (*bprB*), alginate biosynthesis regulator (*algU*), and *farB* (Figure 5B; Supporting Information 1: Table S6). Moreover, comparative genomic analysis of *P. multocida* AH01 revealed high average nucleotide identity with reference strains: 99.71% with swine-derived *P. multocida* HN07 and 99.68% with avian *P. multocida* Pm70 (Supporting Information 3: Figure S2).

### 3.5. Genomic Annotation of *Pasteurella multocida* AH01

**Figure 6:**
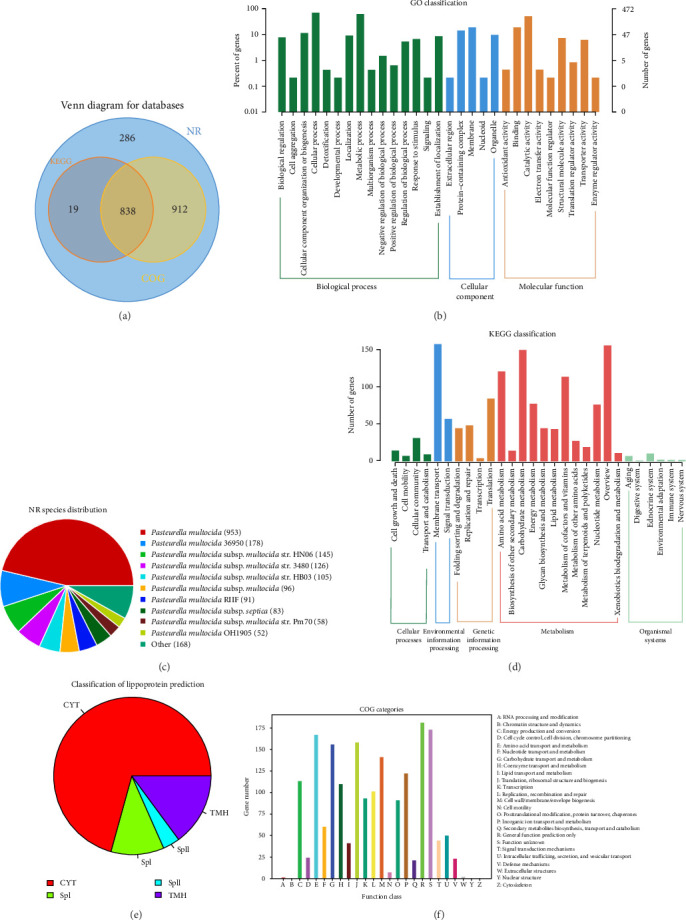
Functional annotation of *Pasteurella multocida* isolate AH01. (A) Database overlap (Venn diagram): KEGG, KOG, and nonredundant (NR) annotations. (B) Gene Ontology (GO) distribution: Histogram showing gene counts (right *y*-axis) and percentages (left *y*-axis) across GO subcategories. Colors denote primary GO categories (BP, CC, and MF). (C) Species homology: Pie chart of top NR BLAST hits (sector area = alignment frequency). (D) KEGG pathway enrichment: Histogram of annotated genes per metabolic pathway. (E) Subcellular localization: Protein class distribution cytoplasmic (CYT), transmembrane helices (TMH), Sec pathway (SpI), and lipoproteins (SpII). (F) Clusters of orthologous groups (COG) functional classification: Histogram of gene counts per orthologous category (color-coded).

Functional annotation assigned 100% of CDSs to the NR database, while coverage in other databases varied: COG (85.03%), KEGG (41.64%), CDD (49.66%), CAZy (1.26%), PHI-base (19%), and GO (22.93%; Supporting Information 1: Table S7). A core set of 838 genes were shared among NR, KEGG, and COG annotations (Figure 6A). Comparative genomic analysis against the NR database annotated 2055 protein-coding genes, with maximum homology to *P. multocida* Pm70 (953 genes) and *P. multocida* 36950 (178 genes; Figure 6C). GO classification assigned 472 genes to 29 subcategories: cellular processes (330 genes) dominated biological processes, membrane components (91 genes) constituted the largest cellular compartment, and catalytic activity (240 genes) represented the predominant molecular function (Figure 6B; Supporting Information 1: Table S8). KEGG pathway mapping of 857 genes highlighted metabolism (848 genes, 98.95%) as the most enriched category, followed by environmental information processing (214 genes) and genetic information processing (180 genes; Figure 6D and Supporting Information 1: Table S9). COG classification categorized 1750 genes into 24 functional groups, with translation machinery (158 genes), amino acid transport/metabolism (167 genes), and carbohydrate transport/metabolism (156 genes) forming the core functional repertoire (Figure 6F and Supporting Information 1: Table S10). CAZy analysis identified 26 genes, predominantly glycosyltransferases (GTs, *n* = 16), along with glycoside hydrolases (GHs, *n* = 5) and auxiliary activities (AAs, *n* = 3; Supporting Information 1: Table S11). Subcellular localization predicted 53.55% cytoplasmic (CYT), 21.89% membrane-associated, and 0.49% secreted proteins (Supporting Information 1: Table S12). Lipoprotein analysis revealed the following subcellular localizations: 70.60% CYT, 10.88% containing signal peptide I (SpI), 3.55% with lipoprotein signal peptide II (SpII), and 14.82% possessing N-terminal transmembrane helices (TMHs; Figure 6E and Supporting Information 1: Table S13).

### 3.6. Virulence and Antibiotic Resistance Gene Profiling

Comparative analysis against the PHI-base revealed that *P. multocida* AH01 exhibit seven distinct virulence phenotype categories and harbors 391 pathogen–host interaction-related genes. Notably, 62.7% (245/391) of these genes correlated with attenuated virulence phenotypes (Supporting Information 1: Table S14). The VFDB serves as a comprehensive repository and analytical platform for bacterial pathogenesis research, containing two-tiered datasets: Set A (experimentally validated virulence determinants with phenotypic evidence) and Set B (bioinformatically predicted virulence-associated elements). Genomic screening of VFDB identified 127 virulence-associated genes in *P. multocida* AH01 exhibiting strict homology to experimentally validated virulence determinants in VFDB Set A, while comparative analysis against Set B revealed 254 putative virulence-associated homologs (Supporting Information 1: Table S15).

AMR profiling of *P. multocida* AH01 identified 47 resistance genes, including those conferring resistance to peptide antibiotics, macrolides, sulfonamides, aminoglycosides, glycopeptides, tetracyclines, and other antibiotics. The associated resistance mechanisms encompassed target alteration, efflux pump systems, and enzymatic inactivation (Supporting Information 1: Table S16).

### 3.7. Comparative Genomic Analysis

**Figure 7:**
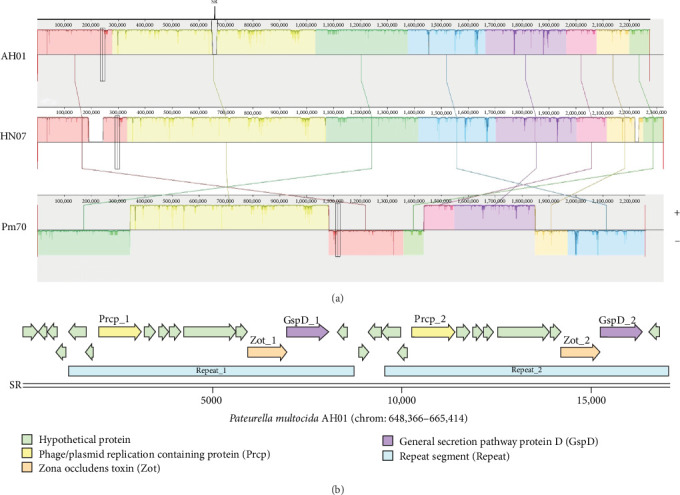
Comparative genomics of *Pasteurella multocida* serogroup F strains and analysis of specific region (SR) in the AH01 genome. (A) Whole-genome alignment of porcine strain AH01, avian strain Pm70, and porcine strain HN07 using progressiveMauve (v2.4.0). Rectangles of similar color show colinear blocks of genes. In the Pm70 panel, the colored blocks labeled “+” indicates genes oriented in the same direction as in the AH01 genome, while blocks labeled “−” indicates genes oriented in the reverse direction to the Pm70 genome. The reduced height of the shading shows areas of low identity within colinear blocks. Genes unique to the AH01 genome (absent in Pm70 and HN07) were marked with a specific region (SR). (B) Schematic diagram of SR gene structure.

Comparative genomic analysis revealed that AH01 exhibit eight large collinear blocks (LCBs) maintaining structural conservation with both HN07 and Pm70 reference genomes, as visualized in the whole-genome alignment (Figure 7A). Notably, we identified a 16.7-kb strain-specific region (SR, chrom: 648366-665414) contains GI_1 and prophage_1, which was absent in the comparator strains. This SR contained two tandem repeats flanking virulence-associated loci encoding Zot and GspD, alongside a phage/plasmid replication protein (Figure 7B). NCBI BLASTn analysis showed the highest nucleotide identity with *P. multocida* D01 (98.25%) and *Actinobacillus pleuropneumoniae* AH2022 (86.95%), with synteny conservation confirmed by Easyfig (Supporting Information 4: Figure S3).

**Figure 8:**
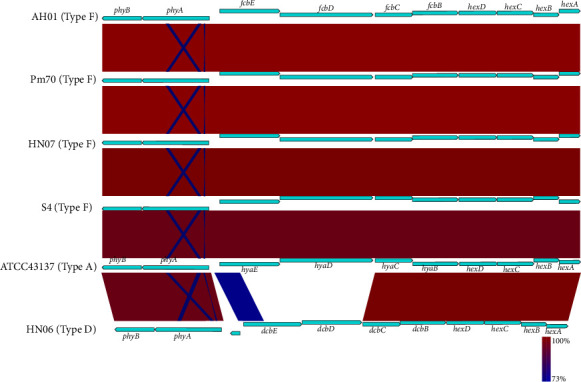
Comparative genomic analysis of the complete capsular biosynthesis locus (cap locus) between AH01 and other *Pasteurella multocida* strains. The AH01 cap locus was aligned with representative strains of different capsular types: Pm70 (Type F), HN07 (Type F), PF17 (Type F), S4 (Type F), ATCC43137 (Type A), HB03 (Type A), and HN06 (Type D). The color code represents the BLASTn identity.

Comparative analysis revealed that the entire capsular locus of AH01 was highly similar to those of avian (Pm70), porcine (HN07), and lagomorph (S4) strains. Furthermore, the entire cap locus of capsular serotype F was most closely related to that of Type A but distinct from those of *P. multocida* capsular serotype D (Figure 8).

**Figure 9:**
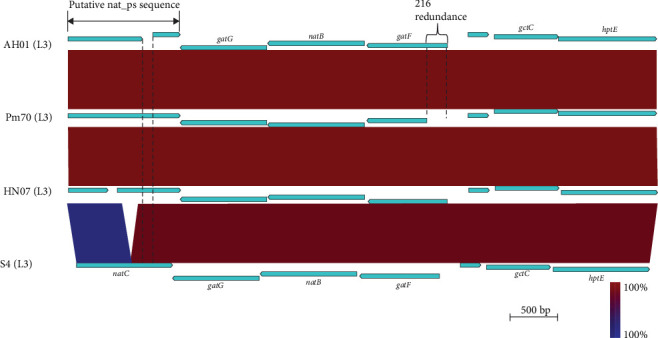
Comparative genomic architecture of lipopolysaccharide (LPS) biosynthesis loci in *Pasteurella multocida* strains AH01, Pm70, HN07, and S4. Comparative analysis of the LPS outer core biosynthesis locus in AH01. The biosynthetic genes required for the assembly of the LPS outer core in AH01 were compared with those of Pm70, HN07, and S4, all of which belong to LPS antigen type L3. Colors indicate percentage identity based on BLASTn analysis.

Comparison of LPS biosynthesis genes with those of other strains showed that the LPS genetic locus in AH01 was also highly conserved in HN07, Pm70, and S4 (Figure 9). Orthologs of the GT genes required for the synthesis of the outer core section of the LPS molecule in Pm70, including *gatG* (pm1139), *natB* (pm1140), *gatF* (pm1141), *gctC* (pm1143), and *hptE* (pm1144), were expectedly identified in AH01 (designated ACLVWN_00090-ACLVWN_00125, correspondingly), while *natC* (pm1138) was the exception. Interestingly, two putative genes (designated ACLVWN_00120-ACLVWN_00125), including a pseudogene (ACLVWN_00125), identified in AH01 were found to have nucleotide homologs to the GT gene *natC* (pm1138) in Pm70. ACLVWN_00120 was highly homologous to the C-terminus of *natC* (pm1138), while pseudo-ACLVWN_00125 was completely matched to the N-terminus (Supporting Information 5: Figure S4), compared with the N-terminus of *natC* (pm1138). Moreover, ACLVWN_00125 represented a pseudogene containing the N-terminal domain with a vacancy at position 760, which resulted in the regrouping of all codons downstream from the deletion point, introducing a premature stop codon (Supporting Information 6: Figure S5). Similarly, pmhn07_0025 had a G→A mutation at position 410, resulting in similar translation termination. Furthermore, an in-frame deletion of 183-bp nucleotide (in positions 559–741) was observed in the *natC* of S4. Notably, AH01 (pmah01_0021), HN07 (pmhn07_0021), and S4 (LCY73_00300) exhibited 216-bp redundant sequences in their N-terminal regions compared to Pm70 *gatF* (pm1141; Figure 9).

## 4. Discussion

The pathogenicity of *P. multocida* serogroup F, which was first identified during an avian cholera outbreak in 1987, has since expanded to include mammalian hosts. Experimental challenges by Jaglic et al. [29] confirmed significant virulence in rabbits and mice, highlighting cross-species transmission risks. Serosurveys detected Type F strains in porcine pneumonia cases as early as 2003 [45], until 2016, Peng et al. [15] characterized a porcine-derived *P. multocida* serogroup F strain with unequivocal clinical manifestations. However, the pathogenic mechanisms and genetic features of porcine-adapted Type F strains are still poorly understood.

To address this gap, we evaluated the pathogenicity of a porcine *P. multocida* serogroup F isolate AH01. Intratracheal challenge induced hyperthermia (40.3–41.5°C) and severe clinical signs (anorexia, lethargy, dyspnea, and orthopnea) within 6 hpi, with mortality commencing at 12 hpi. Necropsy revealed classic *P. multocida* pathology: abundant tracheal frothy exudate, yellowish thoracic effusion, pericardial effusion, and pulmonary congestion/edema with sarcoidosis [29, 31, 46]. Histopathology confirmed fibrinous exudates, hemorrhagic foci, interlobular septal thickening, and pronounced inflammatory cell infiltration-consistent with endotoxin-mediated pulmonary damage during bacterial proliferation [47]. These findings robustly establish AH01 as a virulent porcine pathogen.

Although numerous genomes of *P. multocida* from various serogroups and hosts have been sequenced, complete genomic data for porcine-origin serogroup F strains remains limited. The complete genome sequencing and annotation of strain AH01 enriched existing genomic resources for this species. Our analysis revealed a specific genomic region in AH01 containing genes encoding Zot and GspD. In detailed, Zot, originally identified in *Vibrio cholerae*, increases intestinal permeability by disrupting tight junctions through receptor-mediated signaling [48]. GspD is a core secretin component of T2SS and shows significant homology with secretins such as PilQ and YscC/HrcC. Its N-terminal periplasmic domain likely facilitates interactions with secreted substrates and T2SS-associated proteins [49]. This region also contains a phage/plasmid replication-associated protein and overlaps with GI_1 and prophage_1. As key MGEs, such GIs and prophages contribute significantly to bacterial evolution and pathogenicity by enabling horizontal gene transfer—facilitating the spread of virulence factors like Zot and GspD—and through synergistic regulation of virulence gene expression via prophage lytic–lysogenic switching. These GIs identified in AH01 not only differentiate our strain from less-virulent reference isolates but also provide important molecular insights into its pathogenic mechanisms and evolutionary history.

The pathogenicity of *P. multocida* is associated with diverse virulence factors, including adhesins, dermonecrotic toxin, iron acquisition proteins, sialidases, and outer membrane proteins [27, 50, 51]. A total of 23 such factors have been previously described [52, 53]. In AH01 strain, 16 core virulence genes were identified, encompassing fimbriae and adhesin-related genes (*ptfA*, *fimA*, *hsf-1*, *hsf-2*, and *tadD*), iron metabolism regulators and uptake-related genes (*exbB*, *exbD*, *tonB*, and *fur*), sialidase-encoding gene (*nanH*), outer membrane protein genes (*ompA*, *ompH*, *oma87*, and *plpB*), and superoxide dismutase genes (*sodA* and *sodC*). Iron acquisition systems, particularly the *exbB*-*exbD* operon and fur-mediated regulation, facilitate bacterial survival and infection by scavenging host iron resources [20]. *sodA* and *sodC* are crucial for counteracting oxidative stress during host-pathogen interactions. Furthermore, key outer membrane proteins (OmpA, Omp87, OmpH, and PlpB) were also present, consistent with their established roles in infection [3, 54]. In contrast to the 20 virulence genes reported by Li et al. [55], AH01 lacks *toxA* and *nanB*, but carries *nanH*. Furthermore, comparative analysis confirms the importance of capsule polysaccharides as a key virulence determinant in avian and hemorrhagic septicemia pathogenesis [56], supporting the genotypic profile observed herein.

LPS is a major virulence factor in *P. multocida* [18]. Genomic comparisons revealed that polymorphisms in LPS outer core biosynthetic genes, particularly *natC* and *gatF*, are associated with host adaptation. Rabbit-derived strains (e.g., S4, PF1–PF19) consistently exhibit structural variations such as a 61-amino-acid truncation in *natC* and an N-terminal extension in *gatF*, which are thought to modify LPS structure and promote immune evasion [13, 57]. The conservation of these mutations among rabbit isolates implies strong host-specific selection. Notably, porcine strains AH01 and HN07 showed high sequence similarity in these genes, differing only in *natC* truncation lengths while maintaining identical *gatF* redundancies. In contrast, avian (Pm70) and other porcine strains displayed distinct LPS gene configurations, likely restricting their capacity to effectively colonize rabbits.

## 5. Conclusions

In conclusion, this study identifies *P. multocida* serogroup F strain AH01 as a highly virulent porcine pathogen capable of inducing lethal pneumonia with rapid mortality (≤12 hpi). We present the complete genome sequence of the porcine-adapted serogroup F strain AH01, which harbors a novel 16.7-kb virulence-associated region encoding Zot and GspD proteins likely involved in disrupting epithelial integrity. Key polymorphisms in LPS biosynthetic genes (*natC* and *gatF*) were identified across *P. multocida* serogroup F strains (avian Pm70, porcine AH01, and HN07). Strain AH01 carries a frameshift deletion at *natC* in position 760, resulting a premature stop codon. Both porcine strains (AH01 and HN07) share a 216-bp extension in *gatF* compared to avian Pm70, indicating host-specific or strain-dependent LPS biosynthetic divergence. These findings significantly enhance our understanding of *P. multocida* serogroup F pathogenicity and provide a genetic basis for developing targeted interventions against emerging porcine pasteurellosis.

## Data Availability

All data supporting the findings of this study are included within the paper.

## Supporting Information

Supporting Information 1 Table S1. Primer sequences for identification of *Pasteurella multocida* strain AH01. Table S2. Collaborating strain information. Table S3. Antimicrobial susceptibility test of *Pasteurella multocida* strain AH01. Table S4. General genomic characteristics of *Pasteurella multocida* strain AH01. Table S5. The table of genome island prediction result. Table S6. The table of prophage prediction result. Table S7. Gene annotation ratio statistics. Table S8. Gene Ontology (GO) annotation statistics table. Table S9. Statistical table of KEGG database annotation pathway information. Table S10. Statistical table of Clusters of Orthologous Groups (COG) database annotation function information. Table S11. Carbohydrate-active enzymes (CAzys) summary statistics table. Table S12. Summary table of protein subcellular localization. Table S13. Summary table of lipoprotein prediction results. Table S14. Summary table of PHI phenotype classification. Table S15. Summary table of virulence factor annotation. Table S16. Summary table of drug resistance function annotations.

Supporting Information 2 Figure S1. Isolation and identification of *Pasteurella multocida*. (A) Colonial morphology of the isolated strain on TSA agar supplemented with 5% newborn calf serum. (B) Colonial morphology on blood agar. (C) Gram staining of the isolate under 1000x magnification (scale bar = 10 µm).

Supporting Information 3 Figure S2. Comparative genomic analysis of AH01 with HN07, CIRMBP-0884, CIRMBP-0873, CQ2, HN06, HN02, P1059, PF17, and Pm-3 strain. The inner three rings represent the genome size, GC content, and GC skew of AH01. The outer ten rings (from inside to outside) indicate genomic regions sharing sequence identity with AH01 for each of the compared strains, in the order: Pm70, HN07, CIRMBP-0884, CIRMBP-0873, CQ2, HN06, HN02, P1059, PF17, and Pm-3. This image was generated using the BLAST Ring Image Generator (BRIG; http://brig.sourceforge.net).

Supporting Information 4 Figure S3. Analysis of specific region (SR) in the AH01 genome. BLASTn comparison of SR with *P. multocida* D01 (CP170336.1) and Actinobacillus pleuropneumoniae AH2022 (CP141949.1) visualized with Easyfig (v2.2.5). Color gradient reflects sequence identity (scale: 67%–100%).

Supporting Information 5 Figure S4. Comparison of the C-terminal sequences of the *natC* (pm1138) gene in the lipopolysaccharide (LPS) biosynthesis locus across four *Pasteurella multocida* strains: AH01 (ACLVWN_00120), Pm70 (pm1138), HN07 (pmcn07_0024), and S4 (LCY73_00285). The red box indicates the gap position in AH01 (ACLVWN_00120).

Supporting Information 6 Figure S5: Comparison of the N-terminal sequences of the *natC* (pm1138) genein the lipopolysaccharide (LPS) biosynthesis locus across four *Pasteurella multocida* strains: AH01 (ACLVWN_00125), Pm70 (pm1138), HN07 (pmcn07_0025), and S4 (LCY73_00285). The red box indicates the gap position in AH01 (ACLVWN_00125).
