# Supplementary material for: Pathogenicity and Genomic Characterization of Pasteurella multocida Serogroup F Isolate AH01 From Porcine Pneumonia in China
Source: Transbound Emerg Dis. 2025 Nov 11;2025:9979547. doi: 10.1155/tbed/9979547 (PMC12626689; doi:10.1155/tbed/9979547)
Supplement: Supporting Information 1 — Table S1. Primer sequences for identification of Pasteurella multocida strain AH01. Table S2. Collaborating strain information. Table S3. Antimicrobial susceptibility test of Pasteurella multocida strain AH01. Table S4. General genomic characteristics of Pasteurella multocida strain AH01. Table S5. The table of genome island prediction result. Table S6. The table of prophage prediction result. Table S7. Gene annotation ratio statistics. Table S8. Gene Ontology (GO) annotation statistics table. Table S9. Statistical table of KEGG database annotation pathway information. Table S10. Statistical table of Clusters of Orthologous Groups (COG) database annotation function information. Table S11. Carbohydrate-active enzymes (CAzys) summary statistics table. Table S12. Summary table of protein subcellular localization. Table S13. Summary table of lipoprotein prediction results. Table S14. Summary table of PHI phenotype classification. Table S15. Summary table of virulence factor annotation. Table S16. Summary table of drug resistance function annotations. [file 9979547.f1.docx]

**TABLE S1. Primer sequences for identification of *P. multocida* strain AH01.**

| **Serogroup** | **Gene** | **Primers sequence** | **Product size (bp)** |
| --- | --- | --- | --- |
| All | *KMT1* | F:5′-ATCCGCTATTTACCCAGTGG-3′ | 460 |
|  |  | R:5′-GCTGTAAACGAACTCGCCAC-3′ |  |
| A | *hyaD-hyaC* | F:5′-TGCCAAAATCGCAGTCAG-3′ | 1,044 |
|  |  | R:5′-TTGCCATCATTGTCAGTG-3′ |  |
| B | *bcbD* | F:5′-CATTTATCCAAGCTCCACC-3′ | 760 |
|  |  | R:5′-GCCCGAGAGTTTCAATCC-3′ |  |
| D | *dcbF* | F:5′-TTACAAAAGAAAGACTAGGAGCCC-3′ | 657 |
|  |  | R:5′-CATCTACCCACTCAACCATATCAG-3′ |  |
| E | *ecbJ* | F:5′-TCCGCAGAAAATTATTGACTC-3′ | 511 |
|  |  | R:5′-GCTTGCTGCTTGATTTTGTC-3′ |  |
| F | *fcbD* | F:5′-AATCGGAGAACGCAGAAATCAG-3′ | 851 |
|  |  | R:5′-TTCCGCCGTCAATTACTCTG-3′ |  |
| L1 | *pcgD-pcgB* | F:5′-ACATTCCAGATAATACACCCG-3′ | 1,307 |
|  |  | R:5′-ATTGGAGCACCTAGTAACCC-3′ |  |
| L2 | *nctA* | F:5′-CTTAAAGTAACACTCGCTATTGC-3′ | 810 |
|  |  | R:5′-TTTGATTTCCCTTGGGATAGC-3′ |  |
| L3 | *gatF* | F:5′-TGCAGGCGAGAGTTGATAAACCATC-3′ | 474 |
|  |  | R:5′-CAAAGATTGGTTCCAAATCTGAATGGA-3′ |  |
| L4 | *latB* | F:5′-TTTCCATAGATTAGCAATGCCG-3′ | 550 |
|  |  | R:5′-CTTTATTTGGTCTTTATATATACC-3′ |  |
| L5 | *RmlA-rlmC* | F:5′-AGATTGCATGGCGAAATGGC-3′ | 1,175 |
|  |  | R:5′-CAATCCTCGTAAGACCCCC-3′ |  |
| L6 | *nctB* | F:5′-TCTTTATAATTATACTCTCCCAAGG-3′ | 668 |
|  |  | R:5′-AATGAAGGTTTAAAAGAGATAGCTGGAG-3′ |  |
| L7 | *ppgB* | F:5′-CCTATATTTATATCTCCTCCCC-3′ | 931 |
|  |  | R:5′-CTAATATATAAACCATCCAACGC-3′ |  |
| L8 | *natG* | F:5′-GAGAGTTACAAAAATGATCGGC-3′ | 255 |
|  |  | R:5′-TCCTGGTTCATATATAGGTAGG-3′ |  |

**TABLE S2. Collaborating strain information.**

| **Strain (GenBank Accession Number)** | **Host** | **Serogroup** |
| --- | --- | --- |
| AH01(CP178495.1) | pig | F |
| Pm70(AE004439.1) | avian | F |
| HN07(CP007040.1) | pig | F |
| HN06(CP003313.1) | pig | D |
| CQ2(CP033599.1) | bovine | / |
| PF17(CP112895.1) | rabbit | F |
| Pm-3(CP014618.1) | bovine | A |
| CIRMBP0873(CP020347.1) | rabbit | / |
| CIRMBP0884(CP020345.1) | rabbit | / |
| P1059(CM001581.1) | avian | A |
| HB01(CP006976.1) | bovine | A |
| HB03(CP003328.1) | pig | A |
| Razi-Pm0001(cp017961.1) | bovine | B |
| HN02(CP37865.1) | sheep | / |
| 40540(CP097796.1) | turkey | A |

**TABLE S3. Antimicrobial susceptibility test of *P. multocida* strain AH01.**

| **Drugs** | **Antibacterial circle diameter (mm)** | **Sensitivity** |
| --- | --- | --- |
| penicillin | 27.20 | Resistance |
| ampicillin | / | Resistance |
| Amoxicillin | / | Resistance |
| Amoxicillin-clavulanicacid | 27.54 | Sensitive |
| ceftriaxone | 18.00 | Sensitive |
| cephalothin | 17.84 | Intermediate |
| cefotaxime | 29.34 | Sensitive |
| Oxytetracycline | 15.10 | Sensitive |
| Doxycycline | 24.32 | Sensitive |
| tetracycline | 12.21 | Intermediate |
| streptomycin | 13.18 | Intermediate |
| neomycin | 14.04 | Intermediate |
| Spectinomycin | 18.10 | Sensitive |
| azithromycin | 15.30 | Intermediate |
| erythromycin | 13.00 | Resistance |
| Enrofloxacin | 25.30 | Sensitive |
| ciprofloxacin | 20.38 | Intermediate |
| lincomycin | / | Resistance |
| Florfenicol | 25.84 | Sensitive |
| chloramphenicol | 27.08 | Sensitive |
| polymyxinB | 14.62 | Sensitive |
| trimethoprim | / | Resistance |
| trimethoprim-sulfamethoxazole | / | Resistance |

**TABLE S4. General genomic characteristics of *P. multocida* strain AH01.**

| **Item** | **Number** |
| --- | --- |
| Genome size (bp) | 2,273,743 |
| Total number of sequences | 38,764 |
| Total amount of base data | 377,118,100 |
| Average sequence length | 9,728.57 |
| Number of coding genes | 2,058 |
| G+C content (%) | 40.34 |
| CDS | 2,058 |
| tRNA | 57 |
| 16S rRNA | 6 |
| 23S rRNA | 6 |
| 5S rRNA | 7 |
| ncRNA | 4 |

**TABLE S5. The table of genome island prediction result.**

| **GeneIsland** | **length** | **Gene_Count** |
| --- | --- | --- |
| 1:648,486-667,021 | 18,536 | 31 |
| 2:1,441,483-1,456,943 | 15,461 | 20 |
| 3:1,621,840-1,636,662 | 14,823 | 16 |

**TABLE S6. The table of prophage prediction result.**

| **proPhage** | **length** | **Gene Count** |
| --- | --- | --- |
| 1:645,647-674,556 | 28,910 | 37 |
| 2:1,584,685-1,636,662 | 51,978 | 49 |

**TABLE S7. Gene annotation ratio statistics.**

| **Annotation database** | **Number of genes** | **Annotation ratio (%)** |
| --- | --- | --- |
| CDD | 1,022 | 49.66 |
| COG | 1,750 | 85.03 |
| NR | 2,055 | 99.85 |
| CAzy | 26 | 1.26 |
| PHI | 391 | 19 |
| GO | 472 | 22.93 |
| KEGG | 857 | 41.64 |

**TABLE S8. Gene Ontology (GO) annotation statistics table.**

| **Ontology** | **Term** | **Number** |
| --- | --- | --- |
| biological process | biological regulation | 37 |
|  | cell aggregation | 1 |
|  | cellular component organization or biogenesis | 54 |
|  | cellular process | 330 |
|  | detoxification | 2 |
|  | developmental process | 1 |
|  | localization | 43 |
|  | metabolic process | 298 |
|  | multi-organism process | 2 |
|  | negative regulation of biological process | 7 |
|  | positive regulation of biological process | 3 |
|  | regulation of biological process | 25 |
|  | response to stimulus | 32 |
|  | signaling | 1 |
|  | establishment of localization | 41 |
| cellular component | extracellular region | 1 |
|  | protein-containing complex | 69 |
|  | membrane | 91 |
|  | nucleoid | 1 |
|  | organelle | 46 |
| molecular function | antioxidant activity | 2 |
|  | binding | 91 |
|  | catalytic activity | 240 |
|  | electron transfer activity | 2 |
|  | molecular function regulator | 1 |
|  | structural molecule activity | 35 |
|  | translation regulator activity | 4 |
|  | transporter activity | 30 |
|  | enzyme regulator activity | 1 |

**TABLE S9. Statistical table of KEGG database annotation pathway information.**

| **Pathway Level1** | **Pathway Level2** | **Number** | |
| --- | --- | --- | --- |
| Cellular Processes | Cell growth and death | | 14 |
|  | Cell motility | | 7 |
|  | Cellular community | | 31 |
|  | Transport and catabolism | | 9 |
| Environmental Information Processing | Membrane transport | | 157 |
|  | Signal transduction | | 57 |
| Genetic Information Processing | Folding sorting and degradation | | 44 |
|  | Replication and repair | | 48 |
|  | Transcription | | 4 |
|  | Translation | | 84 |
| Metabolism | Amino acid metabolism | | 120 |
|  | Biosynthesis of other secondary metabolites | | 14 |
|  | Carbohydrate metabolism | | 149 |
|  | Energy metabolism | | 77 |
|  | Glycan biosynthesis and metabolism | | 44 |
|  | Lipid metabolism | | 43 |
|  | Metabolism of cofactors and vitamins | | 113 |
|  | Metabolism of other amino acids | | 27 |
|  | Metabolism of terpenoids and polyketides | | 19 |
|  | Nucleotide metabolism | | 76 |
|  | Overview | | 155 |
|  | Xenobiotics biodegradation and metabolism | | 11 |
| Organismal Systems | Aging | | 7 |
|  | Digestive system | | 1 |
|  | Endocrine system | | 10 |
|  | Environmental adaptation | | 2 |
|  | Immune system | | 1 |
|  | Nervous system | | 1 |

**TABLE S10. Statistical table of Clusters of Orthologous Groups (COG) database annotation function information.**

| **Code** | **Name** | **Number** |
| --- | --- | --- |
| A | RNA processing and modification | 1 |
| C | Energy production and conversion | 113 |
| D | Cell cycle control, cell division, chromosome partitioning | 24 |
| E | Amino acid transport and metabolism | 167 |
| F | Nucleotide transport and metabolism | 60 |
| G | Carbohydrate transport and metabolism | 156 |
| H | Coenzyme transport and metabolism | 110 |
| I | Lipid transport and metabolism | 41 |
| J | Translation, ribosomal structure and biogenesis | 158 |
| K | Transcription | 93 |
| L | Replication, recombination and repair | 101 |
| M | Cell wall/membrane/envelope biogenesis | 141 |
| N | Cell motility | 7 |
| O | Posttranslational modification, protein turnover, chaperones | 91 |
| P | Inorganic ion transport and metabolism | 122 |
| Q | Secondary metabolites biosynthesis, transport and catabolism | 21 |
| R | General function prediction only | 181 |
| S | Function unknown | 173 |
| T | Signal transduction mechanisms | 44 |
| U | Intracellular trafficking, secretion, and vesicular transport | 50 |
| V | Defense mechanisms | 23 |
| W | Extracellular structures | 2 |

**TABLE S11. Carbohydrate-Active enZYmes (CAzy) summary statistics table.**

| **CAZy _Class** | **CAzy** | **Gene_count** |
| --- | --- | --- |
| AA | AA1 | 1 |
| CE | CE1 | 2 |
|  | CE11 | 1 |
|  | CE9 | 1 |
| GH | GH103 | 1 |
|  | GH13 | 1 |
|  | GH3 | 1 |
|  | GH32 | 1 |
|  | GH77 | 1 |
| GT | GT19 | 1 |
|  | GT2 | 1 |
|  | GT25 | 2 |
|  | GT28 | 1 |
|  | GT35 | 1 |
|  | GT4 | 1 |
|  | GT41 | 3 |
|  | GT5 | 1 |
|  | GT51 | 1 |
|  | GT8 | 1 |
|  | GT9 | 3 |

**TABLE S12. Summary table of protein subcellular localization.**

| **Location** | **Count** | **Ratio (%)** |
| --- | --- | --- |
| Cytoplasmic | 1,102 | 53.55 |
| Secreted | 10 | 0.49 |
| Membrane | 450 | 21.89 |
| Total | 2,058 | 100 |

**TABLE S13. Summary table of lipoprotein prediction results.**

| **Class** | **Count** | **Ratio (%)** |
| --- | --- | --- |
| CYT（Cytoplasmic protein） | 1,453 | 70.60 |
| SpI（Signal peptide I） | 224 | 10.88 |
| SpII（lipoprotein signal peptide II） | 73 | 3.55 |
| TMH（N-terminal transmembrane helix） | 305 | 14.82 |
| All | 2,058 | 100 |

**TABLE S14. Summary table of PHI phenotype classification.**

| **Phenotype** | **Gene count** |
| --- | --- |
| chemistry target: sensitivity to chemical | 2 |
| effector (plant avirulence determinant) | 4 |
| increased virulence (hypervirulence) | 21 |
| lethal | 4 |
| Loss of pathogenicity | 26 |
| reduced virulence | 245 |
| unaffected pathogenicity | 89 |

**TABLE S15. Summary table of virulence factor annotation.**

| **DB** | **Total_Proteins** | **Predicted_VF_Proteins** | **Ratio (%)** |
| --- | --- | --- | --- |
| setA | 2,058 | 127 | 6.17 |
| setB | 2,058 | 238 | 11.56 |

**TABLE S16. Summary table of drug resistance function annotations.**

| **ARO name** | **Antibiotic resistance** |
| --- | --- |
| rpoB | rifamycin resistance |
| msbA | nitroimidazole resistance |
| macB, oleC | macrolide resistance |
| Ugd, eptA, OmpA, basS, arnA | peptide resistance |
| TaeA | pleuromutilin resistance |
| tet(35), TxR, tetA(58), tetA(58) | tetracycline resistance |
| cpxA, MexW, LptD, hmrM, CRP, optrA, vatB, CpxR, clbB, dfrA3, smeR, NmcR, MexH, efrA, rsmA, salE, YajC, vgaB | Multiple resistances |
| bcr-1 | bicyclomycin-like resistance |
| AbaF | phosphonic acid resistance |
| sul4 | sulfonamide resistance |
| vanL, vanTG, vanRE, vanHA, vanRO, vanRL, vanHM, golS, H-NS | glycopeptide resistance |
| farB | antibacterial free fatty acids |
| RanA | aminoglycoside resistance |
| msbA | nitroimidazole resistance |
