# Supplementary figures and images for: Pathogenicity and Genomic Characterization of Pasteurella multocida Serogroup F Isolate AH01 From Porcine Pneumonia in China
Source: Transbound Emerg Dis. 2025 Nov 11;2025:9979547. doi: 10.1155/tbed/9979547 (PMC12626689; doi:10.1155/tbed/9979547)

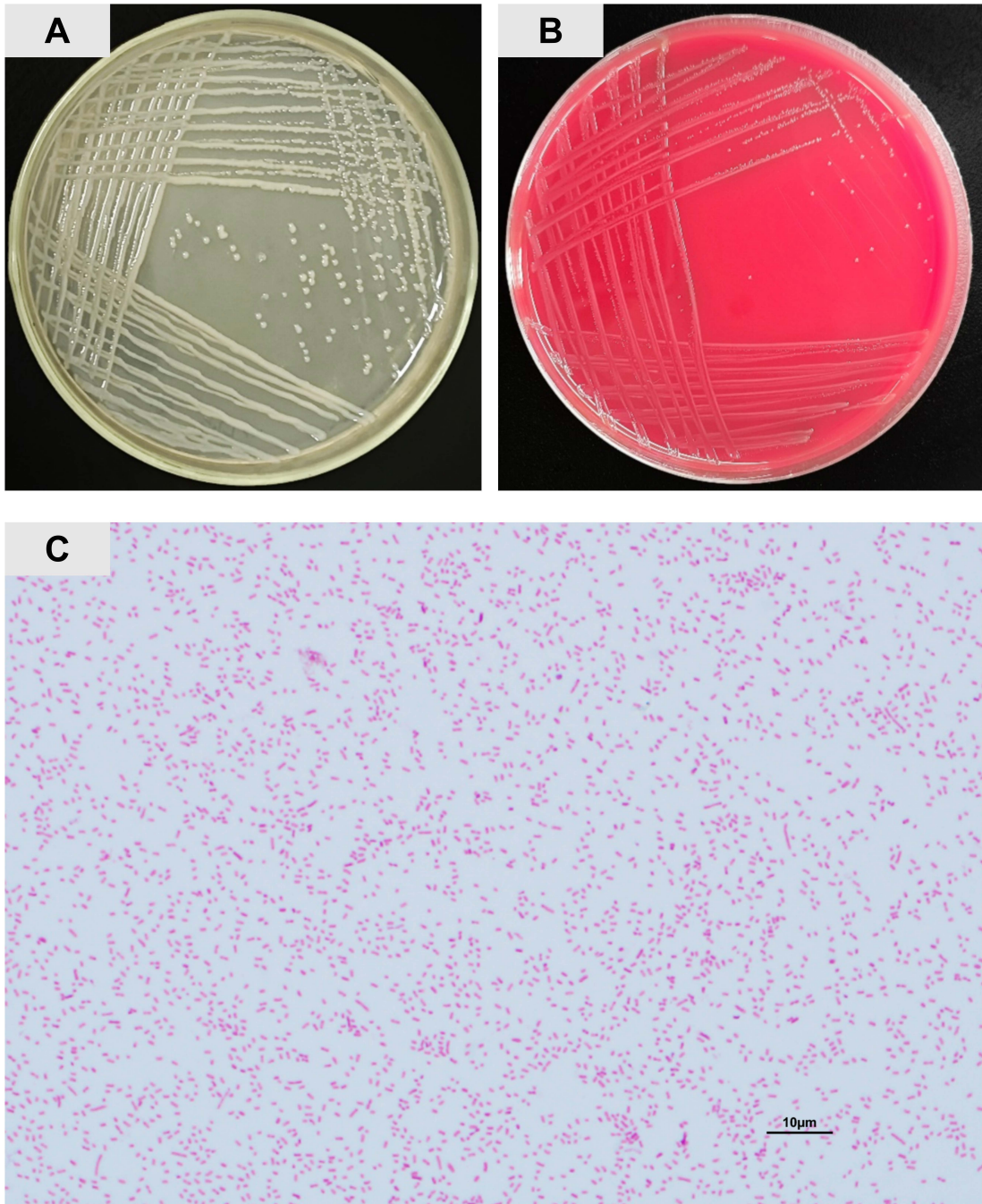

**FIGURE S1**

Supplement: Supporting Information 2 — Figure S1. Isolation and identification of Pasteurella multocida. (A) Colonial morphology of the isolated strain on TSA agar supplemented with 5% newborn calf serum. (B) Colonial morphology on blood agar. (C) Gram staining of the isolate under 1000x magnification (scale bar = 10 µm). [file 9979547.f2.pdf]

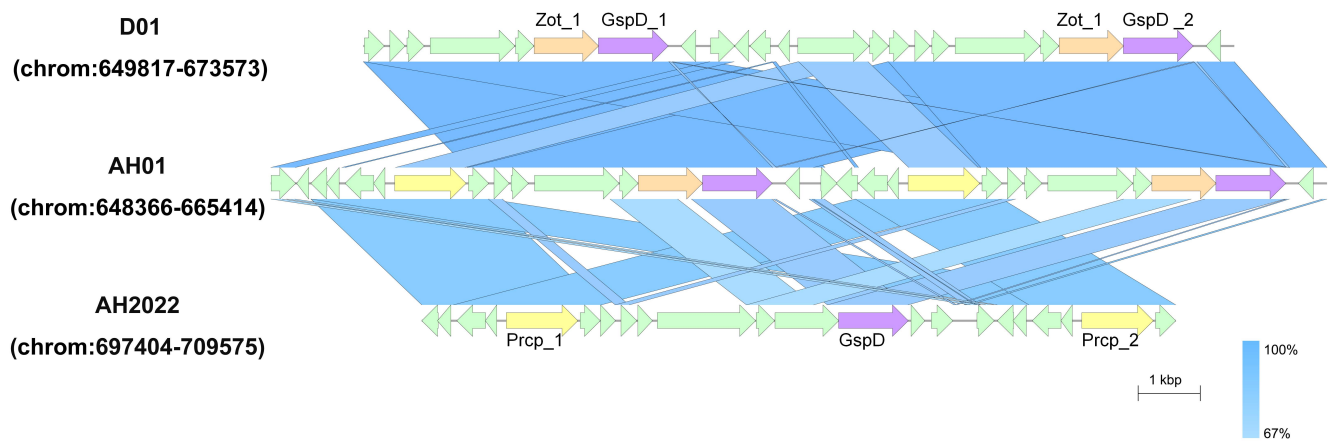

**FIGURE S3**

Supplement: Supporting Information 4 — Figure S3. Analysis of specific region (SR) in the AH01 genome. BLASTn comparison of SR with P. multocida D01 (CP170336.1) and Actinobacillus pleuropneumoniae AH2022 (CP141949.1) visualized with Easyfig (v2.2.5). Color gradient reflects sequence identity (scale: 67%–100%). [file 9979547.f4.pdf]

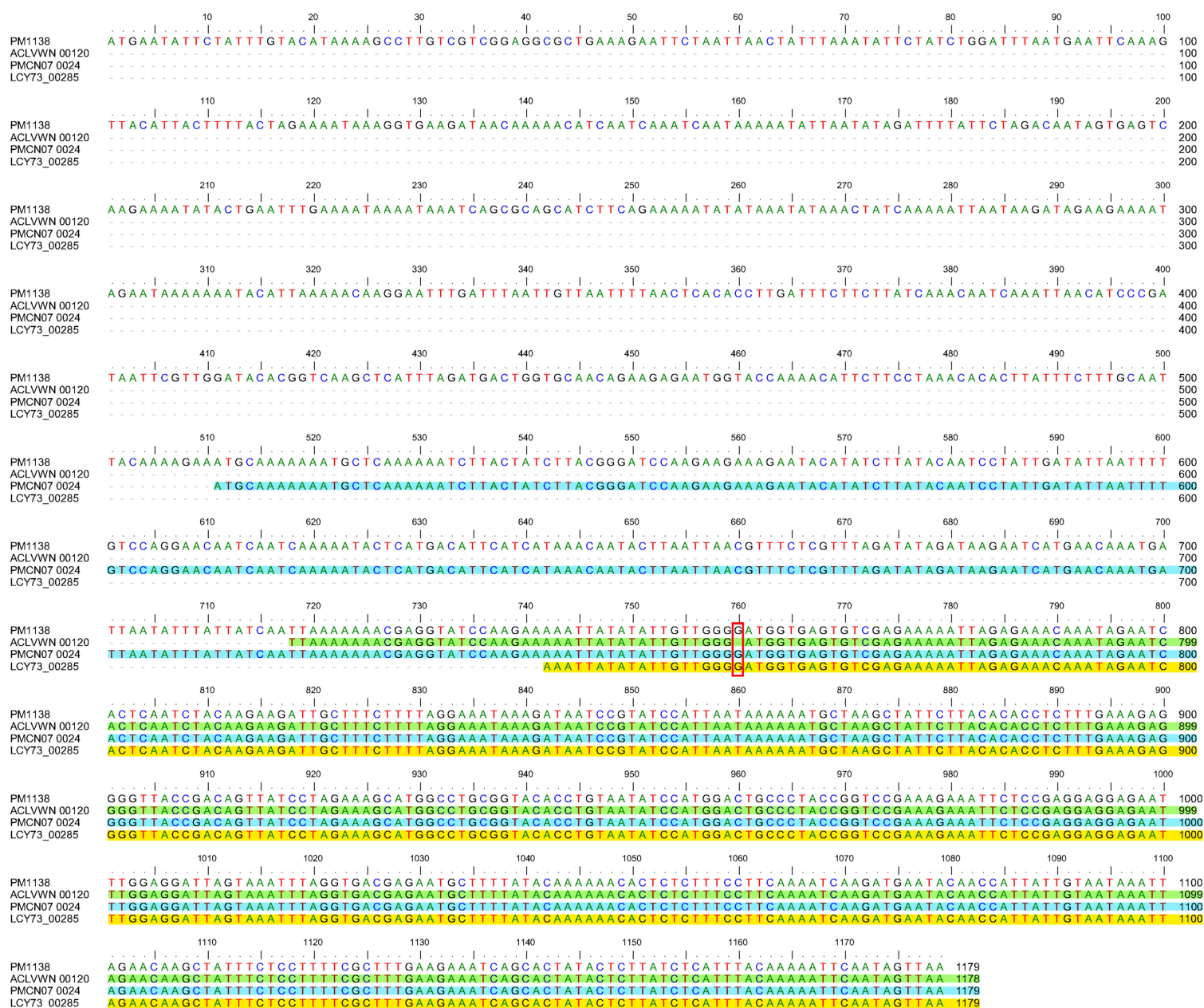

FIGURE S4

Supplement: Supporting Information 5 — Figure S4. Comparison of the C-terminal sequences of the natC (pm1138) gene in the lipopolysaccharide (LPS) biosynthesis locus across four Pasteurella multocida strains: AH01 (ACLVWN_00120), Pm70 (pm1138), HN07 (pmcn07_0024), and S4 (LCY73_00285). The red box indicates the gap position in AH01 (ACLVWN_00120). [file 9979547.f5.pdf]

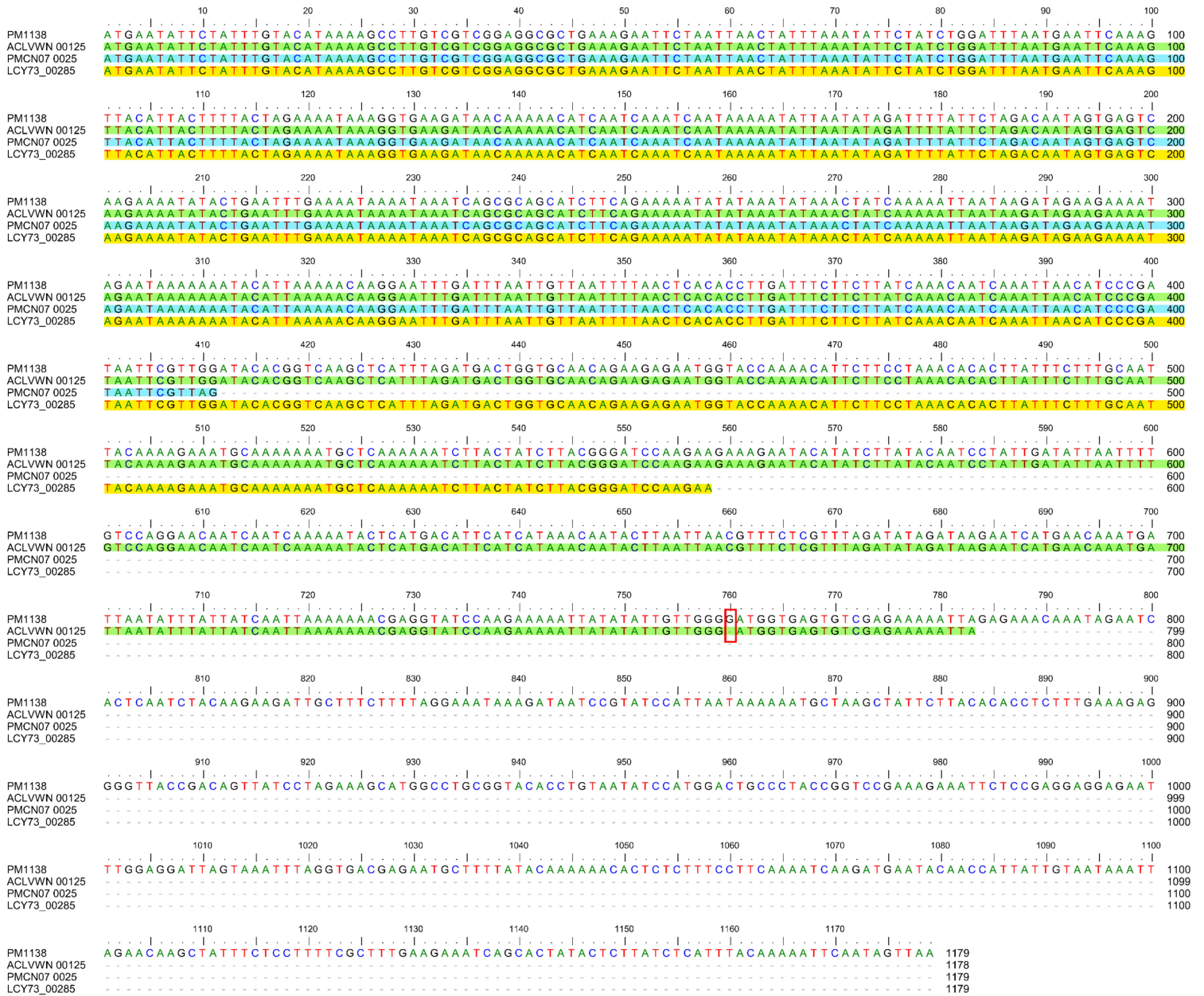

FIGURE S5

Supplement: Supporting Information 6 — Figure S5: Comparison of the N-terminal sequences of the natC (pm1138) genein the lipopolysaccharide (LPS) biosynthesis locus across four Pasteurella multocida strains: AH01 (ACLVWN_00125), Pm70 (pm1138), HN07 (pmcn07_0025), and S4 (LCY73_00285). The red box indicates the gap position in AH01 (ACLVWN_00125). [file 9979547.f6.pdf]
