# Supplementary material for: Pathogenicity and Genomic Characterization of Pasteurella multocida Serogroup F Isolate AH01 From Porcine Pneumonia in China
Source: Transbound Emerg Dis. 2025 Nov 11;2025:9979547. doi: 10.1155/tbed/9979547 (PMC12626689; doi:10.1155/tbed/9979547)
Supplement: Supporting Information 3 — Figure S2. Comparative genomic analysis of AH01 with HN07, CIRMBP-0884, CIRMBP-0873, CQ2, HN06, HN02, P1059, PF17, and Pm-3 strain. The inner three rings represent the genome size, GC content, and GC skew of AH01. The outer ten rings (from inside to outside) indicate genomic regions sharing sequence identity with AH01 for each of the compared strains, in the order: Pm70, HN07, CIRMBP-0884, CIRMBP-0873, CQ2, HN06, HN02, P1059, PF17, and Pm-3. This image was generated using the BLAST Ring Image Generator (BRIG; http://brig.sourceforge.net). [file 9979547.f3.pdf]

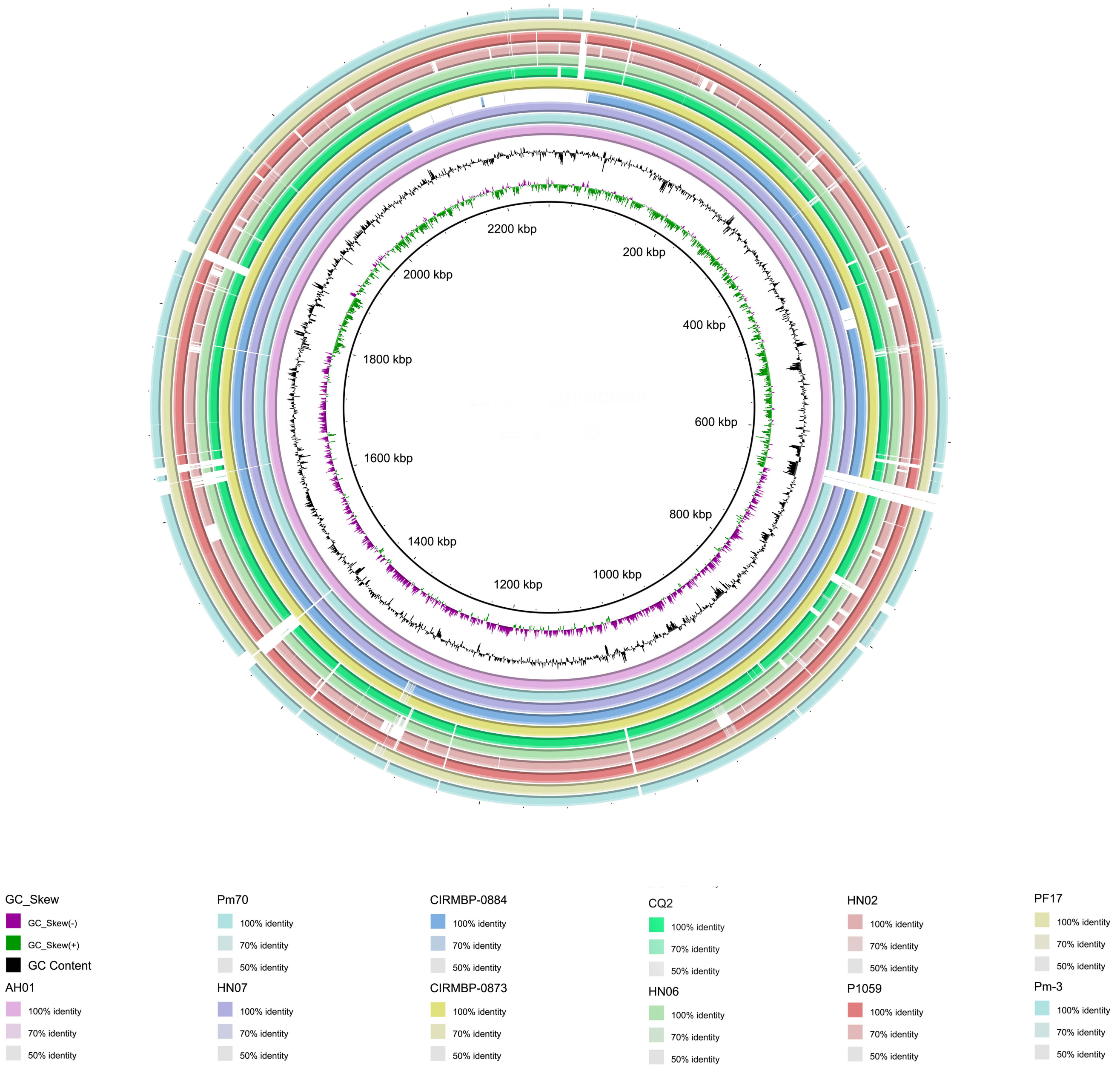

2200 kbp
200 kbp
2000 kbp
400 kbp
1800 kbp
600 kbp
1600 kbp
800 kbp
1400 kbp
1000 kbp
1200 kbp
GC_Skew
GC_Skew(-)
GC_Skew(+)
GC Content
AH01
100% identity
70% identity
50% identity
Pm70
100% identity
70% identity
50% identity
HN07
100% identity
70% identity
50% identity
CIRMBP-0884
100% identity
70% identity
50% identity
CIRMBP-0873
100% identity
70% identity
50% identity
CQ2
100% identity
70% identity
50% identity
HN06
100% identity
70% identity
50% identity
HN02
100% identity
70% identity
50% identity
P1059
100% identity
70% identity
50% identity
PF17
100% identity
70% identity
50% identity
Pm-3
100% identity
70% identity
50% identity

FIGURE S2
